# Group-Personalized Regression Models for Predicting Mental Health Scores From Objective Mobile Phone Data Streams: Observational Study

**DOI:** 10.2196/10194

**Published:** 2018-10-22

**Authors:** Niclas Palmius, Kate E A Saunders, Oliver Carr, John R Geddes, Guy M Goodwin, Maarten De Vos

**Affiliations:** 1 Institute of Biomedical Engineering Department of Engineering Science University of Oxford Oxford United Kingdom; 2 Department of Psychiatry University of Oxford Oxford United Kingdom; 3 Oxford Health National Health Service Foundation Trust University of Oxford Oxford United Kingdom; 4 Sleep and Circadian Neuroscience Institute Nuffield Department of Clinical Neurosciences University of Oxford Oxford United Kingdom

**Keywords:** behavioral features, depression, geolocation, group-personalized model, interindividual variability, mental health, mental illness, objective behavioral markers

## Abstract

**Background:**

Objective behavioral markers of mental illness, often recorded through smartphones or wearable devices, have the potential to transform how mental health services are delivered and to help users monitor their own health. Linking objective markers to illness is commonly performed using population-level models, which assume that everyone is the same. The reality is that there are large levels of natural interindividual variability, both in terms of response to illness and in usual behavioral patterns, as well as intraindividual variability that these models do not consider.

**Objective:**

The objective of this study was to demonstrate the utility of splitting the population into subsets of individuals that exhibit similar relationships between their objective markers and their mental states. Using these subsets, “group-personalized” models can be built for individuals based on other individuals to whom they are most similar.

**Methods:**

We collected geolocation data from 59 participants who were part of the Automated Monitoring of Symptom Severity study at the University of Oxford. This was an observational data collection study. Participants were diagnosed with bipolar disorder (n=20); borderline personality disorder (n=17); or were healthy controls (n=22). Geolocation data were collected using a custom Android app installed on participants’ smartphones, and participants weekly reported their symptoms of depression using the 16-item quick inventory of depressive symptomatology questionnaire. Population-level models were built to estimate levels of depression using features derived from the geolocation data recorded from participants, and it was hypothesized that results could be improved by splitting individuals into subgroups with similar relationships between their behavioral features and depressive symptoms. We developed a new model using a Dirichlet process prior for splitting individuals into groups, with a Bayesian Lasso model in each group to link behavioral features with mental illness. The result is a model for each individual that incorporates information from other similar individuals to augment the limited training data available.

**Results:**

The new group-personalized regression model showed a significant improvement over population-level models in predicting mental health severity (*P*<.001). Analysis of subgroups showed that different groups were characterized by different features derived from raw geolocation data.

**Conclusions:**

This study demonstrates the importance of handling interindividual variability when developing models of mental illness. Population-level models do not capture nuances in how different individuals respond to illness, and the group-personalized model demonstrates a potential way to overcome these limitations when estimating mental state from objective behavioral features.

## Introduction

One key research area in computing and mental health is finding relationships between objective markers of user behavior and mental state. Objective markers in this context may include physical activity levels, geographic movements, interaction with social networks, sleep quality and circadian regularity, and interaction with technology, among others. Commonly, these markers may be recorded continuously from smartphones, which provide easy access to an array of sensors that can provide such data in a completely passive way, although wearable devices and other sensors may also be used.

Objective behavioral markers have been explored in a number of previous studies. Physical activity level was one of the first behavioral markers to be widely studied. For example, Wielopolski et al [1] found significantly lower levels of physical activity in patients with acute unipolar depression than in healthy controls and that physical activity correlated with improvement in symptoms in 19 patients with depression. In contrast, Wang et al [2] found that the levels of activity in students were negatively correlated with self-rated loneliness scores, but not with self-ratings of depression. Use of technology, especially mobile phones, has also been studied, with Saeb et al [3] reporting that both frequency of phone usage and total time spent interacting with the phone correlated with levels of depression in a community cohort of 21 individuals, with more depressed individuals interacting more with their phones. Mehrotra et al [4] also reported that the way users interacted with their phones, such as the number of notifications responded to and the time taken to respond, strongly correlated with depression scores from 25 individuals. Another promising behavioral data source is the geographic movement of individuals, which indicates activity on a higher level with potentially greater accuracy than physical activity recorded through accelerometry. While people may not carry their phones during all physical activity, they are likely to carry them when moving across larger geographic distances. Among the earliest work on using geolocation for mental state estimation, Grünerbl et al [5-6] demonstrated that it is possible to use geolocation-derived features to detect episodes in patients with bipolar disorder. Saeb et al [3] further showed that features derived from geolocation data correlated with depression levels in individuals recruited over the internet; the results were later replicated with a sample of students [7].

The previous work summarized above has focused mainly on finding population-level models [3,6,7] or correlations [2,4] that link measured behavioral markers to mental state. Specifically, most studies either attempt to classify groups of patients by condition or perform regression to estimate the patient’s mental state (usually using patient self-ratings—a limitation explored further in the Discussion). Population-level models use all available data from a given population to link the mental states of individuals to their behavioral features. This is also the case in the popular approach of classifying patient groups based on their behavioral symptoms. It is implicitly assumed that the same set of features will have discriminative power across the whole population. While this is a promising approach, it is widely accepted that natural variability in the usual behavioral patterns of different individuals, or differences in their behavior for a given level of illness, are major limitations of these models. Mohr et al referred to this as the “curse of variability” [8]. A recent review by Berrouiguet et al [9] also highlighted the need to move toward a personalized approach for developing digital tools, especially in mental healthcare. While agreeing that different individuals may have different models that link their behavior to their mental states, how best to define such individual models is still an open question. Training “fully personalized” models on any available training data for each individual (for example, by asking the individual to provide objective data and questionnaire responses for a number of weeks) would in theory produce the most accurate model for that individual because it would eliminate any interindividual variability. Grünerbl et al [5] used this approach to identify mental state in patients with bipolar disorder from geolocation-derived features. Similarly, Canzian and Musolesi [10] reported correlations calculated for individual participants between geolocation-derived features and daily self-reported depression scores in 28 participants who provided at least 20 usable data points. Significant correlations were shown between depression scores and the maximum distance between any 2 locations over the last 14 days for 18 of the 28 participants; this again demonstrates the utility of geolocation as a predictor for depression. However, in practice, this approach is limited by the amount of data that would be required from each individual to train the model, may not generalize to unseen states, and will likely overfit available data, leading to reduced out-of-sample performance.

A compromise between population-level models and fully personalized models is to create groups of individuals with similar characteristics [11]. New individuals can then be allocated to an existing group based on their similarity to individuals in that group. If limited training data are available from new individuals, then their data can be combined with data from similar individuals. In a large study of over 18,000 people, Servia-Rodríguez et al [12] found correlations between most of the available demographics (age, gender, occupation, etc) and smartphone-recorded behavioral data. This means that another way of achieving personalized models is by conditioning on available demographics, but this approach is limited by the level of detail in the recorded demographic data. Hong et al have also previously demonstrated that demographic features poorly predict sensor data similarity [13]. Likely more subtle subgroups exist in the population, with similar correlations between behavioral data and mental state, but these groups may not be identifiable by explicit demographic features alone. The challenge is finding these subgroups, which can be thought of as “behavioral phenotypes” within the population. In addition to providing improved models of patient health, understanding the characteristics of these phenotypes may help develop our understanding of how to classify or subclassify mental illness [14].

Personalization by finding similar individuals in the population has long been considered in activity recognition from sensor data [11]. For example, Lane et al [15] developed, and Abdullah et al [16] extended, an approach for finding groups of individuals based on the similarity of their demographics, lifestyle, and sensor data. An advantage of this method is that it does not require any calibration data for new individuals. However, clustering on distributions of sensor data and not on relationships between sensor data and model output (in the present case, mental state) may miss important differences or similarities among individuals.

This paper proposes a novel, data-driven approach toward group-personalized regression. We describe an extension to classical linear regression whereby groups of individuals who exhibit similar linear models linking objective behavioral data and mental state are automatically identified. This model’s ability to identify groups of similar individuals is demonstrated by comparing traditional population-level regression with the novel approach for the estimation of self-reported levels of depression from objective geolocation-derived features.

## Methods

### Data Collection

Data were collected from participants in the Automated Monitoring of Symptom Severity (AMoSS) study at the University of Oxford [17,18]. The AMoSS study was approved by the Research Ethics Committee of the East of England (reference 13/EE/0288), and all participants provided written informed consent. During the AMoSS study, a range of behavioral data were collected from patients diagnosed with bipolar disorder and borderline personality disorder as well as healthy control individuals without any symptoms of mental disorder. All participants were screened by an experienced psychiatrist using the Structured Clinical Interview for the Diagnostic and Statistical Manual of Mental Disorders, Fourth edition. Objective behavioral data were collected from a custom Android-based app as well as other wearable devices. The app recorded individuals’ activity levels, geographic movements (described below), light exposure, and social interaction. We also collected physiological data during some parts of the study, as presented by Carr et al [19,20]. More details about the study design are available in our previous work [17].

### Mental State Reporting

Participants self-reported their mental state throughout their participation in the study, using a variety of clinically validated questionnaires, administered weekly. One questionnaire, the 16-item Quick Inventory of Depressive Symptomatology (QIDS) Self-Report questionnaire [21], was used to assess the level of depression in individuals. This questionnaire asks 16 questions assessing depression based on clinical diagnostic criteria and provides a single score from 0 (no symptoms of depression) to 27 (severe symptoms of depression). A score over 10 is considered a suitable threshold for clinically significant depression. A simple custom mood questionnaire was also administered daily on the app, which Tsanas et al have shown to correlate well with QIDS [22,23].

### Location Data Overview

One of the key sources of behavioral data that can be easily recorded through a mobile phone is the individual’s geographic movements. We described in detail the collection, noise-removal, processing, and feature extraction from geolocation data in our previous work [18], wherein we demonstrated that several features can clearly discriminate between nondepressed and depressed weeks in patients with bipolar disorder.

Features were extracted from preprocessed geolocation data. In total, 10 features described in Table 1 were extracted and used in this paper, with full details available in our previous work [18]. All features were calculated on full calendar weeks of data (Monday to Sunday).

### Data Inclusion

For the analysis presented here, it is important to have multiple data points available for each individual. For this reason, only participants who provided ≥6 weeks of geolocation data with associated QIDS scores were included in the analysis. A total of 59 participants provided the required minimum of 6 labeled weeks of data. Demographic characteristics of the included participants are shown in Table 2, which also shows summary statistics of the data available for analysis. Healthy control participants had the lowest mean QIDS scores and least variability. Participants with borderline personality disorder had the highest mean QIDS scores and also the highest variability, with participants with bipolar disorder between the two.

### Standard Population-Level Regression Model

Previous studies estimating continuous mental health severity from objective markers [3], commonly work with standard linear regression models [24] of the form: y=βx^T^+µ.

In our case, x would be a vector of the geolocation-derived features in Table 1, and *y* is the predicted QIDS score. This regression model therefore forms the core baseline model for comparison of the group-personalized model described in the following section. Linear models as given above are prone to overfitting the available training data resulting in poor out-of-sample performance. One well-known method to reduce the overfitting of the model is the Lasso by Tibshirani [25], which adds an ℓ_1_ regularization term to select only the most important features to include in the model. In its Lagrangian dual form [26], the Lasso is parameterized by λ. When λ is large, many (or all) of the coefficients of β will be reduced to exactly zero.

**Table 1:** Summary of features extracted from preprocessed geolocation data.

Feature name	Description
Location variance	A measure of the variance in the location coordinates visited.
Number of clusters	The number of unique locations visited.
Entropy of locations	The information-theoretic entropy calculated on the proportion of time spent in each of the locations visited.
Normalized entropy	The entropy of locations feature normalized by dividing by the log of the number of location visited, resulting in a feature ranging between 0 and 1, which is less correlated with the number of clusters feature.
Home stay	The percentage of time that the individual is recorded at home.
Transition time	The percentage of time that the individual is recorded traveling between locations.
Total distance	The total distance traveled by the individual.
Diurnal movement	A measure of the diurnal regularity in the movements of the individual, calculated from the power in sinusoids fitted to the data with periods around 24 hours.
Diurnal movement on normalized coordinates	Similar to the diurnal movement feature, but calculated on normalized coordinates, making it less sensitive to the different distances that individuals may travel.
Diurnal movement on distance from home	Similar to the diurnal movement and diurnal movement on normalized coordinates features, but calculated on the single dimensional distance of the current location coordinates from the home location of the individual.

**Table 2:** Demographic and data characteristics of participants included in the analysis.

Characteristic		Healthy controls (n=22)		Bipolar disorder patients (n=20)		Borderline personality disorder patients (n=17)		Total (n=59)	
**Gender, n**									
	Male		7		7		1		15
	Female		15		13		16		44
Age, median (IQR^a^)		42 (12)		44 (20)		38 (9.75)		41 (15.75)	
Body mass index, median (IQR)		24 (5.37)		27 (4.22)		31 (10.25)		26 (8.50)	
Weeks of data per participant, median (IQR)		17 (15.0)		16 (19.5)		20 (24.5)		19 (19.75)	
QIDS^b^ mean, median (IQR)		2 (1.92)		5 (6.74)		14 (4.67)		4 (9.96)	
QIDS range, median (IQR)		3 (2.07)		7 (6.64)		10 (7.34)		5 (7.52)	

^a^IQR: interquartile range.

^b^QIDS: Quick Inventory of Depressive Symptomatology.

**Figure 1:**
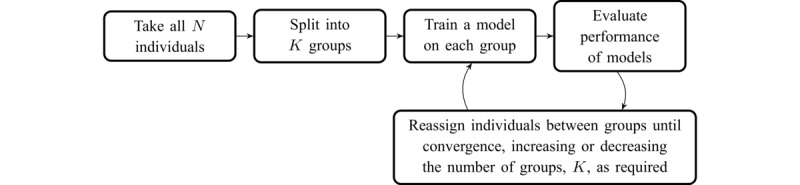
High-level overview of the group-personalized model.

### Group-Personalized Regression Model

Here, we will introduce a method for splitting the total population into groups of individuals that have similar relationships between their geolocation-derived features and their QIDS scores. Each group will be represented by a Lasso model where the regression coefficients β and offset μ will differ between subsets. This group-personalized regression model has been developed to improve regression performance by finding models in subgroups that fit individuals better than the population-level model trained across all available individuals, while not training an individual model for each subject.

Figure 1 depicts the high-level framework of the group-personalized model used to find groups of individuals that have similar regression models linking mental states and behavioral features. During initialization, all available individuals are randomly split into groups, and a Lasso regularized linear regression model is trained for each group. The performance of all individuals under the model for each group is then evaluated, and individuals are reassigned between groups as appropriate. Note that the number of groups is dynamic and optimized as the model is run. This process is repeated to convergence. Using Lasso regularized linear regression as the core of the group-personalized model allows identification of the most relevant subset of features for each group.

In a practical realization of the model depicted in Figure 1, a framework that combines the predictive model and clustering is required. The Dirichlet process (DP) provides a suitable framework [27]. A DP mixture model splits individuals into distinct groups, trains a model on individuals in each group, and then allows individuals to switch groups until clustering is optimized. One feature of the DP is that the number of clusters does not need to be specified in advance, and individuals may create new clusters if they do not fit well into existing ones.

The DP is a Bayesian model where any well-specified generative model can be used as the model for individual clusters (with the requirement that model priors can be sampled and that the joint likelihood can be calculated).

Park and Casella [28] have previously presented a Bayesian version of the Lasso. In this work, we extend the Bayesian Lasso to operate over multiple individuals and apply a DP prior to provide clustering of individuals based on the relationship between their behavioral features and their levels of self-reported depression.

**Figure 2:**
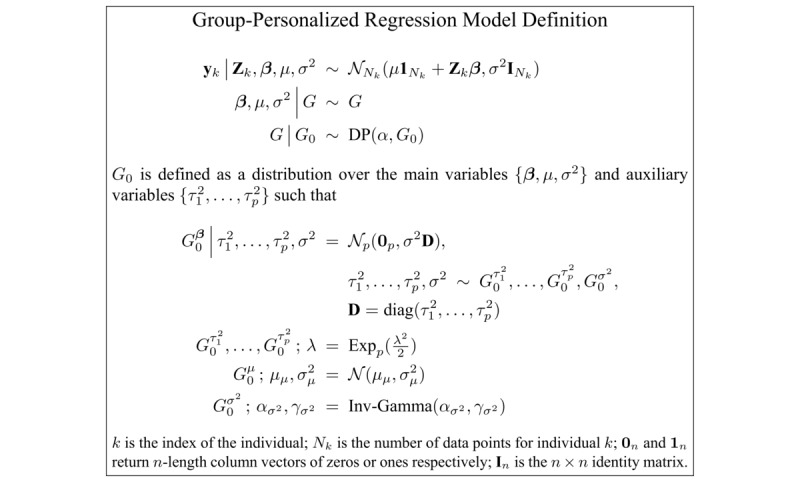
Formal definition of the group-personalized regression model.

The full generative form of the group-personalized regression model is given in Figure 2. Free parameters in the model are the α concentration parameter of the DP; the λ regularization parameter of the Lasso model for each group; the μ_μ_ and σ_μ_^2^ priors of the μ variable (the regression offset); and the α_σ_^2^ and γ_σ_^2^ priors of the σ^2^ variable (the noise in the model). The α concentration parameter and the λ regularization parameter were both optimized by a grid search maximizing the likelihood of the model’s joint probability. Parameter values of λ=0.4 and α=0.0001 were found optimal and are used to generate the results presented here. Other free priors have much less impact on the model and were set to μ_μ_=0; σ_μ_^2^=10; α_σ_^2^=1; and γ_σ_^2^=1.

**Figure 3:**
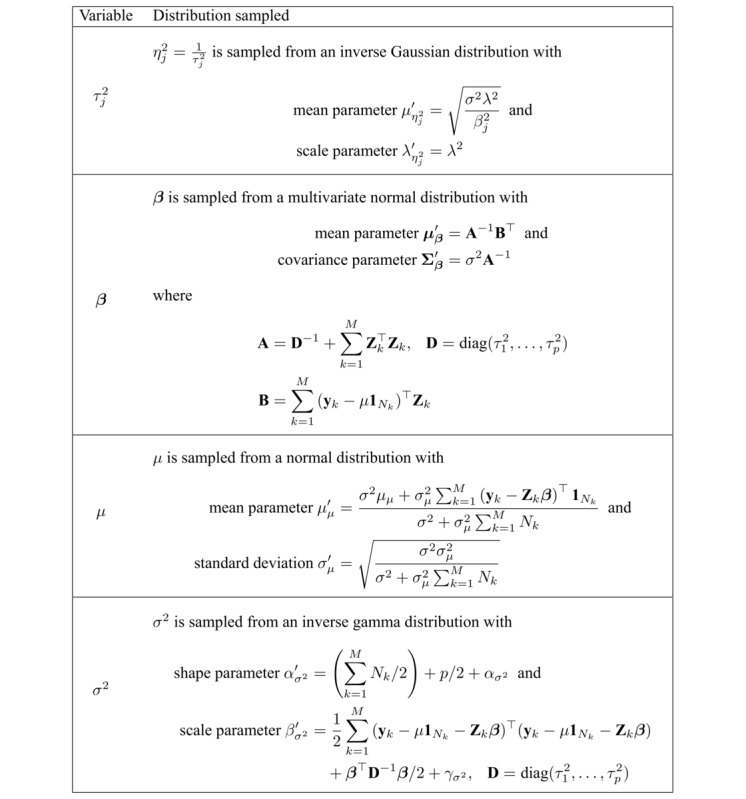
Gibbs sampler distributions for variables in the group-personalized regression model.

The group-personalized regression model defined in Figure 2 can be sampled using a standard implementation of Algorithm 8 by Neal [27,29]. In each iteration of sampling the DP, individual grouped model variables are Gibbs sampled from the distributions given in Figure 3.

### Model Evaluation

#### Evaluation Framework

This study’s aim was to predict QIDS scores from geolocation-derived features and to compare the performance of population-level, fully personalized, and group-personalized regression models for this purpose. For this reason, several models described below have been implemented to present comparative results.

Most models described in the following sections were tested in a “leave-one-participant-out” framework where each participant is left out and the relevant model is trained on data from other relevant participants (as described for each model in turn).

In practice, fully personalized and group-personalized models generally require a certain level of calibration data (behavioral features and QIDS scores). To simulate this, in most models, a subset of data from the start of the recording for the left-out test individual were included in the model’s training (but were excluded when evaluating test performance). The number of weeks of calibration data included in the model training is defined as the first half of the available data for the test individual up to a maximum of 8 weeks. For example, for an individual who provided 10 weeks of data, the first 5 weeks are used as calibration data included in model training and the last 5 weeks are used for evaluation. For an individual who provided ≥16 weeks of data, the first 8 weeks are used as calibration data and all remaining data are used for evaluation.

In all cases except the clustering model, standard Bayesian Lasso models by Park and Casella [28] were trained on the calibration data for the left out individual and all data from other relevant individuals. The Bayesian Lasso model was trained with regularization parameter *λ*=0.4 (the same value found optimal for running the group-personalized model) for 1000 iterations. Because the Bayesian Lasso is implemented using a Gibbs sampler, this leads to 1000 samples of model coefficients. The model’s performance was evaluated in each iteration by applying the Bayesian Lasso with the sampled coefficients on the evaluation data for the left out individual. The mean absolute error (MAE) of the estimated QIDS scores and self-reported values was calculated in each iteration, and overall performance was evaluated as the mean MAE over all iterations. Improvements in performance were compared using a single-tailed paired *t* test of the mean MAE over all iterations for each individual.

#### Population-Level Model

The population-level model was tested by training a Bayesian Lasso model on the calibration data from the test individual and all data available from all other individuals.

#### Group-Personalized Model

The group-personalized model was introduced by first finding groups of individuals in the population that have similar models linking their geolocation-derived features with their QIDS scores. To find these groups, the group-personalized regression model was run using Neal’s Algorithm 8 for 5000 iterations with all data available from all individuals.

To evaluate the performance of the optimal groups found using the group-personalized model, a Bayesian Lasso model was trained on the calibration data from the test individual and all data available from other individuals in the same group.

Note that if an individual ends up in a group containing just himself or herself, then the group-personalized model will be trained just on the calibration data from that individual, so that it reduces to the fully personalized model described below.

#### Group-Personalized Model with Clusters Allocated Using Calibration Data

The group-personalized model tested retrospectively, as described above, is useful to demonstrate principles of the model’s operation and the clinical relevance of the groups found.

To apply the model prospectively, only calibration data from new individuals must be used to allocate them into one of the groups. To test this, each individual was left out in turn, and separate Bayesian Lasso models were trained for each group. For the group that the left-out individual was originally assigned to, the Bayesian Lasso model was trained on that group’s remaining individuals (if any).

QIDS scores were estimated from the calibration data for the left-out individual using the model trained on each of the groups. The mean MAE of the estimated QIDS scores using the model for each group was evaluated, and the individual was allocated to the group that provided optimal performance.

To provide the final prediction, a new Bayesian Lasso model was trained using the calibration data from the left-out individual and all data from other individuals assigned to the allocated group.

#### Fully Personalized Model Using All Available Data

The literature [5,6,30] commonly uses cross-validation over all available data points from an individual to demonstrate a “personalized” model. This was implemented using random subsampling of data from each individual, where a Bayesian Lasso model was trained using 80% of data randomly selected from the test individual, with results presented on estimation of the remaining 20%. This split was repeated 10 times.

#### Fully Personalized Model Using Calibration Data Only

While the fully personalized model using all available data is commonly presented in the literature, it may not provide a fair representation of the model’s accuracy in practice because it does not demonstrate how well the model will generalize when trained with limited calibration data.

As an alternative, the fully personalized model using only calibration data was tested by training a Bayesian Lasso model on just the calibration data from the test individual.

#### Clustering Model

A comparative model was tested using the clustering method described by Lane et al [15] and Abdullah et al [16]. To provide a comparative result, only the part of the method that clusters individuals by similarity of their feature values was included. The clustering method by Lane et al and Abdullah et al used a locality-sensitive hashing method known as random projection [31] as a similarity measure of feature values between all pairs of individuals. The random projection method samples random values in the feature space and calculates the distance from these random values to extracted feature values for each individual. By repeating this process multiple times, features from similar individuals will commonly be closest to the same randomly sampled values. Lane et al and Abdullah et al used the resulting similarity matrix to condition an online boosting classification algorithm. Because the group-personalized model concerns regression rather than classification, this was replaced with a regression equivalent [32].

Because the clustering model does not use the relationship between behavioral features and model output, all available data from all individuals, including the test individual, were included when assessing similarity.

## Results

### Extracted Feature Properties

**Figure 4:**
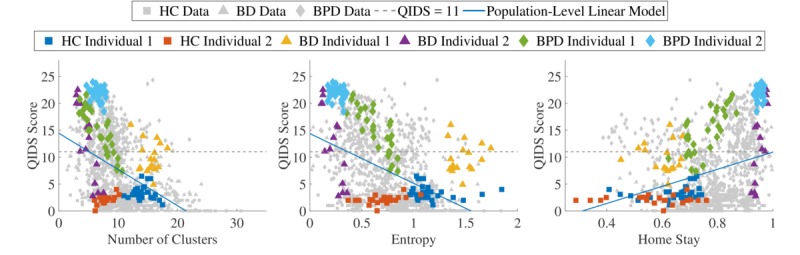
All available data for 3 of the geolocation-derived features, with the data from 6 individuals highlighted, 2 from each cohort in the study. (HC: healthy control, BD: bipolar disorder, BPD: borderline personality disorder, QIDS: Quick Inventory of Depressive Symptomatology).

Raw feature values calculated from the geolocation data for included participants are shown in Figure 4 for 3 selected features (the number of locations visited on the left; entropy in the middle; and the percentage of time recorded at home on the right); highlighted are 6 participants (2 from each cohort). Standard population-level linear regression models predicting the QIDS score from all available data for each of the features individually are shown overlaid. The general trends follow what is expected from the literature and has been presented in previous work. More depressed individuals tend to visit fewer locations and stay at home more. As a measure of variability in the locations visited, entropy tends to be lower in more depressed individuals, indicating that depressed individuals have less regularity in their routines.

Clearly, however, these trends are weak, and the features contain a high level of noise. Models trained over all available data (such as the population-level linear models shown in Figure 4) would therefore tend to estimate depression poorly on behavioral features.

The 6 participants highlighted in Figure 4 indicate that data from specific individuals tended to occur in clusters within the whole dataset. In some cases, such as the green borderline personality disorder individual, personalizing the model using the number of clusters visited should far outperform the population-level model.

### Model Evaluation

**Figure 5:**
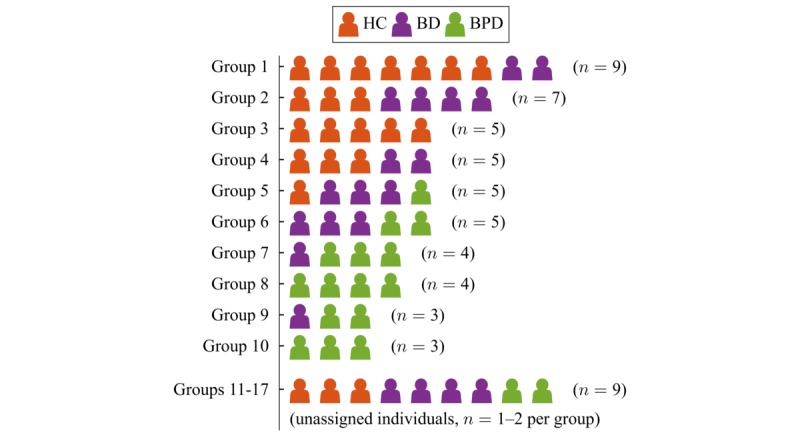
Allocations of individuals to different groups, showing the cohort of each individual. (HC: healthy control, BD: bipolar disorder, BPD: borderline personality disorder).

The group-personalized regression model was run over all individuals as described above. In total, 17 groups were found, each containing between 1 and 9 individuals. Figure 5 shows how individuals were allocated to these groups. Each group is shown as a row, with the shading of the markers indicating how many individuals are from each cohort in that group. Groups 1-4 are predominantly healthy control individuals, together with participants with bipolar disorder who exhibit low variability in their QIDS scores. Groups 5 and 6 are predominantly individuals with bipolar disorder, and groups 7-10 are predominantly individuals with borderline personality disorder, all displaying greater variability in their QIDS scores. To groups 11-17, 9 individuals were assigned, with only 1 or 2 individuals in each group. These individuals have been shown as unassigned because they did not fit well into any of the other groups, and therefore solid conclusions cannot be drawn. As more individuals become available for analysis, these individuals would likely be assigned to larger groups.

The number of groups found (17 for 59 participants) indicates the high level of variability in the relationships between behavioral data and mental state for individuals in the study. The number of groups may also be affected by properties of the DP prior on smaller sample sizes. Asymptotically, the DP exhibits a “rich-gets-richer” property, where larger clusters tend to become larger [33]. This also has the side effect that with more data points, the number of clusters relative to the number of samples decreases (more precisely, the expected number of clusters grows logarithmically with the number of data points, proportional to the concentration parameter in the DP prior). With fewer data points, none of the clusters are yet large enough to sufficiently attract data points. This means that as more data become available to train the model, the relative number of clusters should decrease, thus improving the model’s stability.

These findings imply some overlap between healthy control individuals and individuals with bipolar disorder. This fits with the informal observation that individuals with bipolar disorder exhibit normal mood when well. By contrast, there is very little overlap between healthy control and borderline personality disorder group membership. This finding broadly aligns with other subjective measures of mental state, sampled much more frequently than weekly, which show a gradient of abnormal mood where healthy control<bipolar disorder<borderline personality disorder [19,20]. How more frequent sampling would combine with geolocation data is an important future question.

**Figure 6:**
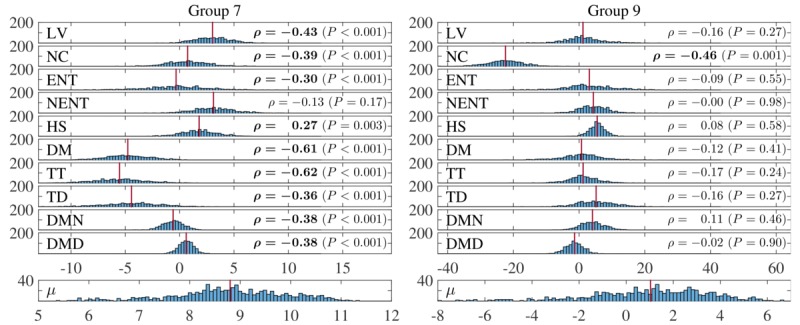
Sampled coefficient values for features in 2 groups found by the group-personalized model. (LV: location variance; NC: number of clusters; ENT: entropy of locations; NENT: normalized entropy; HS: home stay; DM: diurnal movement; TT: transition time; TD: total distance; DMN: diurnal movement on normalized coordinates; DMD: diurnal movement on distance from home).

One of the key advantages of finding similar groups of individuals within the population is that models for each group found may indicate different characteristics of patient subgroups. This can be explored by inspecting the coefficient values sampled for each group. For this, a Bayesian Lasso model was run on all individuals allocated to each group. This results in the sampled coefficient values shown in Figure 6 for groups 7 and 9 in Figure 5.

The group on the left in Figure 6 is characterized mainly by the diurnal movement, transition time, and total distance features, while the group on the right is characterized by the number of clusters visited. In both cases, the other features are effectively removed from the model. Pearson correlation coefficients of each feature with the reported QIDS scores for individuals in the group are shown on the right of each feature. Statistically significant correlation coefficients (*P*<.01) are shown in bold. This shows that the group-personalized model tends to pick out the most correlated features for each group, although this may not necessarily be the case as two features with low correlations may be predictive when combined in a regression model.

 Inspection of the model coefficients in each of the clusters found indicate that they all model different characteristics of the range of relationships between behavioral data and QIDS scores. Most groups are also characterized by ≤3 main features, indicating the interpretability of the models found.

Performance of the different models tested across all individuals is summarized in Table 3. Results are presented as MAE, mean (SD) for all models. Overall estimation accuracy is increased from the population-level model by using both fully personalized and group-personalized models. Optimal results are achieved with the fully personalized model, trained using subsamples of all available data. This is not surprising since the full range of values is likely to be included in the model’s training. The group-personalized model with individuals allocated to their optimal groups improves over the population-level model and is similar to the fully personalized model. A single-tailed paired *t* test confirms that the performance improvement using the group-personalized model with optimized clusters from the population-level model is significant (*P*<.001). The group-personalized model with individuals assigned to groups using only their calibration data also performs significantly better than the population-level model. It also improves slightly on the fully personalized model trained only on the same calibration data, but the improvement is only mildly significant. Performance of the fully personalized model trained using only calibration data indicates the difficulty of generalizability of a model trained using limited data. The clustering method by Lane et al [15] and Abdullah et al [16] improves on the population-level model for bipolar disorder and borderline personality disorder participants, but does not perform better overall. This is can be explained by the method’s entire basis on similarity of input features, not on the similarity of the relationship between the input features and QIDS scores.

**Figure 7:**
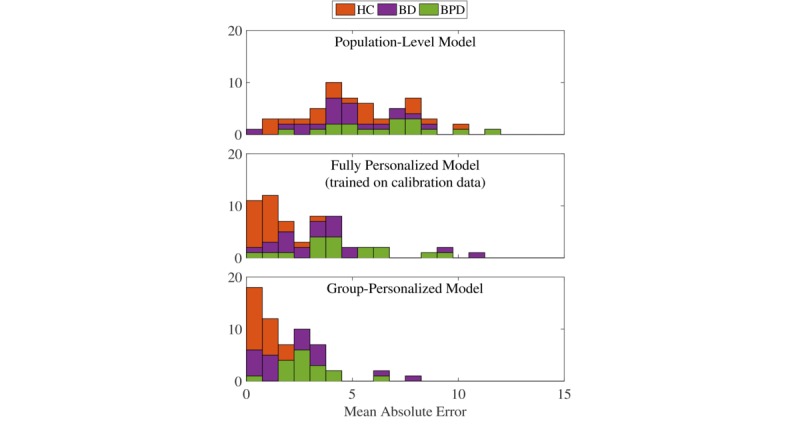
Mean absolute error of Quick Inventory of Depressive Symptomatology score estimation using 3 of the models in Table 3. (HC: healthy control; BD: bipolar disorder; BPD: borderline personality disorder).

Figure 7 shows the distributions of performance under 3 of the models tested: the top row shows the MAE of estimations made using the population-level model; the second row shows the MAE of estimations made using the fully personalized model; and the bottom row shows the MAE of estimations made using the group-personalized model. In all 3 graphs, bars are shaded in proportions corresponding to the cohort of individuals in that bar. Individuals with borderline personality disorder tend to perform worst under the population-level model.

**Table 3:** Mean absolute error of Quick Inventory of Depressive Symptomatology score estimation.

Model	HC^a^, mean (SD)	BD^b^ patients, mean (SD)	BPD^c^ patients, mean (SD)	Overall, mean (SD)	Significance of reduction in overall mean absolute error compared to reference models	
					Population-level model (*P* value)	Fully personalized model trained on calibration data (*P* value)
Population-level model	4.86 (2.54)	4.74 (2.07)	6.43 (2.58)	5.27 (2.48)	—^d^	—
Fully personalized model using cross-validation validation over all data points	0.80 (0.76)	2.27 (1.44)	3.05 (1.67)	1.94 (1.60)	<.001	<.001
Fully personalized model trained on calibration data	1.06 (0.75)	3.67 (2.60)	4.38 (2.43)	2.90 (2.49)	<.001	—
Clustering based on Lane et al [15] and Abdullah et al [16]	5.33 (3.11)	4.50 (2.30)	6.15 (2.88)	5.29 (2.82)	—	—
Group-personalized model with optimized clusters	0.83 (0.52)	2.30 (1.96)	2.82 (1.28)	1.90 (1.60)	<.001	<.001
Group-personalized model with clusters allocated using calibration data	0.86 (0.46)	3.30 (3.09)	3.75 (2.07)	2.52 (2.47)	<.001	.02

^a^HC: healthy control.

^b^BD: bipolar disorder.

^c^BPD: borderline personality disorder.

^d^Not applicable.

The best performance under the group-personalized model is from healthy control participants, who showed quite variable performance under the population-level model. However, this should be viewed with caution because healthy control participants also tended to show very low QIDS scores with very little variation, as demonstrated in Table 2, meaning that a model may just estimate a constant low value. Similarly, some participants with bipolar disorder also perform very well under the group-personalized model, but as shown in Figure 5, some individuals with bipolar disorder were allocated with healthy control individuals, indicating that they may also have relatively constant low QIDS scores. The remaining individuals with bipolar disorder and borderline personality disorder clearly showed great improvement under the group-personalized model.

## Discussion

### Principal Findings

Our previous work demonstrated the utility of using geolocation-derived features to classify weeks of depression in participants with bipolar disorder. In this paper, we investigated to what extent those features can be used as proxy for the level of depression in subjects with bipolar disorder and borderline personality disorder. We have shown that using a population-based estimate is suboptimal because variability in behavioral patterns between subjects is too high, as shown in Figure 4. While fully personalizing a model for each individual might make sense, doing so requires too much data to be practicable in a prospective fashion and provides little clinical insight into how behavior changes with varying levels of depression since every subject follows a different model. We introduce a group-personalized model as an alternative for personalization where subgroups of individuals that exhibit similar relationships between their behavioral data and mental states are automatically identified. This leads to plausible groupings since most healthy control individuals and individuals with bipolar disorder and borderline personality disorder have been assigned to different groups, reflecting the relationship between different disease categories and different behavioral patterns. However, further validation with more data is needed to assess whether these are “optimal” groupings.

While groupings provide insights, the key challenge for applying group-personalized models in practice remains the determination of which group to allocate new individuals to. In the results above, up to 8 data points (pairs of behavioral features and actual QIDS scores) were used to allocate each individual to a group based on the performance of QIDS score estimation using the model for each group. This provided a significant improvement over the population-level model and a smaller, but still mildly significant, improvement over the fully personalized model trained on the same data used for calibration. In practice, the main advantage of grouping might be to enable better understanding of patient characteristics. While the fully personalized model may take any form, the group-personalized model is restricted to a known set of behavioral phenotypes. With enough individuals available to train models, an exhaustive set of behavioral phenotypes can be obtained. Matching individuals to groups based purely on the predictive performance of the extracted groups on calibration data, as done here, is a naïve approach, and using other indicators of similarity, such as demographics or similarity of sensor data, may improve group-matching when only limited calibration data are available.

The fact that a high number of groups relative to the number of individuals were found suggests that there is indeed a large amount of interindividual variability between subjects. Having access to larger datasets might also increase the number of subjects belonging to each group. With the current dataset, groups with clearly different behavioral patterns could be identified, as shown in Figure 6. Significant improvements in performance of estimating levels of depression from objective, geolocation-derived features were shown, demonstrating further the appropriateness of the groupings, but also the utility of using geolocation as an objective marker for mental health.

### Quick Inventory of Depressive Symptomatology Estimation Model

The presented model uses standard linear regression as the core model within groups. This assumes a linear relationship between the model’s predictor(s) and output. Other authors, for instance, Abdullah et al [30], have used more advanced methods such as support vector regression in a similar application, which enables nonlinearity in the relationship between the behavioral features and output variable of interest to be modeled. In the present model, nonlinearity could be modeled using a generalized linear model in place of the standard linear regression. However, the individuals highlighted in the features shown in Figure 4 do not indicate that nonlinearity is a major limitation, but rather variability in the output. For this reason, incorporating nonlinearity may exacerbate any overfitting of the model to the training data available.

### Limitations

While the results presented here demonstrate the utility of group-personalized models of behavior to improve regression performance—which might be a very useful approach beyond this application—a number of important limitations need to be discussed.

First, a limitation of this work, in common with many previous studies of objective markers of mental health, is reliance on and comparison with subjective proxies of mental state. In this work, the patient-reported QIDS questionnaire was used to train and evaluate models. Similar patient-reported questionnaires have been used in most previous work. Some studies use clinician-reported measures, but these are still fundamentally subjective proxies and are usually not available at a high sample rate. While this is an accepted limitation of the current work, as more longitudinal data become available for analysis, the properties of the behavioral phenotypes found may in turn help inform our understanding of mental illness.

The model presented here also assumes that individuals always remain in the same group. In reality, individuals may exhibit temporal variability in their response to illness. For example, in some individuals, improvements in mental state may follow different models to deterioration, or the same individual may have different relationships with behavioral features during different episodes.

The main area of interest in using objective markers to monitor levels of mental illness is the transition between states. Detailed investigation of state transitions compared with stable states may provide useful data about which variations in behavior are normal for an individual in remission (as in bipolar disorder) and which variations significantly correlate with the onset of illness episodes. Separating the two will always be crucial for predictive models to perform adequately. A limitation of the data used to train the models presented in this work is that most individuals did not exhibit both stability and variability in illness. Indeed, most individuals were relatively stable in their levels of depression. Again, as more data become available, more complex analysis can be performed.

### Conclusions

This paper has demonstrated the limitations of using population-level models to estimate levels of mental illness from behavioral features. Population-level models do not account for natural interindividual variability in how individuals’ behavior changes in response to mental illness such as depression. On the other end of the spectrum, fully personalized models built using training data only from specific individuals limits interpretation into clinical phenotypes.

Group-personalized models were therefore presented as a way to augment limited training data available for an individual with data from a group of other individuals who have a similar relationship between their behavior and mental state. Predicting levels of self-reported depression from geolocation-derived features demonstrated the model’s appropriateness. Several previous studies have shown the need for personalized modeling for mental health applications due to the high noise levels in behavioral data, as also demonstrated in this work. While there is a clear advantage in using group-personalized models over population-level models, further work must validate these models. Optimal group allocation remains an open question, but the value in the interpretability of the grouped models has been demonstrated. As further data are collected, the utility of the model is expected to increase because more refined models can be inferred from the groups of individuals found.

## Abbreviations

AMoSS: automated monitoring of symptom severity
BD: bipolar disorder
BDP: borderline personality disorder
DP: Dirichlet process
HC: healthy control
MAE: mean absolute error
QIDS: 16-item Quick Inventory of Depressive Symptomatology, Self-Report version
